# Infective Endocarditis by *Moraxella* Species: A Systematic Review

**DOI:** 10.3390/jcm11071854

**Published:** 2022-03-27

**Authors:** Petros Ioannou, Konstantinos Alexakis, Stella Baliou, Diamantis P. Kofteridis

**Affiliations:** Department of Internal Medicine & Infectious Diseases, University Hospital of Heraklion, 71110 Heraklion, Greece; a.konstantin91@gmail.com (K.A.); stellabaliou@gmail.com (S.B.); kofterid@uoc.gr (D.P.K.)

**Keywords:** endocarditis, systematic review, *Moraxella*

## Abstract

*Moraxella catarrhalis* is the most clinically relevant species among *Moraxella* spp. For decades, it was considered to be part of the normal human flora in the upper respiratory tract. However, since the late 1970s, considerable evidence has proposed that *M. catarrhalis* is an important pathogen in the human respiratory tract. Even though Infective Endocarditis (IE) is rarely caused by *Moraxella* spp., these infections can be problematic due to the lack of experience in their management. The aim of this study was to systematically review all published cases of IE by *Moraxella* spp. A systematic review of PubMed, Scopus and Cochrane library (through 8 December 2021) for studies providing epidemiological, clinical, microbiological data as well as treatment data and outcomes of IE by *Moraxella* spp. was performed. A total of 27 studies, containing data for 31 patients, were included. A prosthetic valve was present in 25.8%. Mitral valve was the most commonly infected site. Fever, sepsis and embolic phenomena were the most common clinical presentations. Cephalosporins, aminoglycosides, aminopenicillins and penicillin were the most commonly used antimicrobials. Overall mortality was 12.9%.

## 1. Introduction

*Moraxella* species are Gram-negative cocci, belonging to the family *Moraxellaceae* that also includes *Acinetobacter* and *Psychrobacter* [1,2]. The most clinically relevant microorganism from this genus is *M. catarrhalis*. This microorganism was first described about a century ago and was proposed by Sir William Osler as the cause of his own terminal pneumonia [3]. After some taxonomic changes, the species was changed from *Neisseria catarrhalis* to *Branhamella catarrhalis* and finally to *M. catarrhalis* [1]. For decades, this microorganism was considered to be part of the normal human flora in the upper respiratory tract; however, since the late 1970s, considerable evidence suggested that *M. catarrhalis* is an important and common pathogen in the respiratory tract in humans [4,5,6,7].

Colonization by *M. catarrhalis* has only highly been detected in humans, and it depends on age. For example, only up to 5% of healthy adults are colonized by *M.* catarrhalis [8,9]. On one hand, in infants, colonization by *M. catarrhalis* is far more common, with rates that even approach 100% [1,10]. On the other hand, even in adults, colonization may be frequent in specific populations. For example, in patients with chronic obstructive pulmonary disease (COPD), *M. catarrhalis* may be isolated relatively frequently. Based on this, colonization of COPD patients by *M. catarrhalis* may be associated with a very high risk of COPD exacerbation [11]. Nowadays, it is established that clinical disease by *M. catarrhalis* most commonly consists of otitis media, lower respiratory tract infection in COPD patients, pneumonia in older adults, nosocomial respiratory tract infections, sinusitis and, more rarely, bacteremia [1]. Interestingly, other members of the *Moraxella* genus, such as *M. lacunata* or *M. nonliquefaciens,* are less frequently observed and described; however, they do have pathogenic potential in humans.

Infective Endocarditis (IE) is an uncommon infection that is associated with significant morbidity and mortality [12,13]. For example, in a recent study in Europe, in-hospital mortality for patients with IE was 17% [14]. In another recent study in Scotland, 30-day and one-year mortality were estimated at about 14% and 30%, respectively [15]. IE is classically caused by Gram-positive microorganisms, such as Staphylococci, Enterococci and Streptococci. However, there are cases of IE caused by Gram-negative bacteria [12,13]. Even though it may be uncommon, IE by Gram-negative bacteria can be problematic, since there is lack of clinical experience with this entity; thus, there is lack of data and guidelines on its treatment [12]. Thus, studies that provide information on the clinical characteristics of IE by Gram-negative species would be valuable, as they could shed light on these rare clinical entities. Interestingly, even though there are scarce data of IE by *Moraxella* spp. in the literature, a review adequately summarizing all available evidence on the topic is lacking, with the exception of some case reports with literature review [16,17].

This study aimed to systematically review all cases of IE by *Moraxella* spp. in the literature and describe the epidemiology, microbiology, clinical characteristics, treatment and outcomes of this rare infection.

## 2. Materials and Methods

### 2.1. Data Search

For this review, we adopted the Meta-analysis of Observational Studies in Epidemiology (MOOSE) guidelines that are more appropriate for systematic reviews, assessing epidemiological studies, but the study also conforms to the Preferred Reporting Items for Systematic reviews and Meta-Analyses (PRISMA) guidelines [18,19]. Eligible studies were identified through search of PubMed, Scopus and Cochrane Library with the following text-words: (((Neisseria or Branhamella) AND catarrhalis) OR Moraxella) AND endocarditis. Day of last search was 8 December 2021.

### 2.2. Study Selection

Studies were included in analysis if they met the following criteria: (1) published in English; (2) reporting data on patients’ clinical characteristics, microbiology, treatment and outcomes. However, studies with the following criteria were excluded from the analysis: (1) secondary research papers (e.g., reviews), editorials and papers not reporting results on primary research; (2) studies not in humans; (3) studies not in English; (4) studies not referring to IE by *Moraxella* spp. Two investigators (P.I., K.A.) using Abstrackr [20] independently reviewed the titles and abstracts of the resulting references; then they retrieved and rescreened the full text publications of potentially relevant articles. Study selection was based on consensus. Reference lists of included studies were searched for relevant articles. In the case where the investigators were unable to access a full-text publication, attempts were made to communicate with the study authors in order to kindly provide the full text.

### 2.3. Outcomes of Interest

The primary outcomes of the study were to record data on the following: (a) epidemiology of patients with IE by *Moraxella* spp. and (b) patients’ outcomes. Secondary outcomes were to record data on (a) the exact site of infection, (b) the patients’ clinical characteristics, (c) antimicrobial susceptibility and (d) their treatment. 

### 2.4. Data Extraction and Definitions

Data from each eligible study were extracted by two investigators (P.I. and K.A.). The extracted data included study type, year of publication and country; patients’ demographic data (age and gender); patients’ relevant medical history (previous cardiac surgery or cardiac valve replacement, time after cardiac valve replacement); infection data and microbiology (infection site, isolated strains, site of microorganism isolation, presence of complications, presence of embolic phenomena); treatment administered for IE; and outcomes (i.e., cure or death). Data on microbiology and association of mortality with the index infection were reported according to the study authors. Diagnosis of IE was confirmed by the investigators based on information provided by the authors and the modified Dukes’ criteria if the diagnosis was at least possible (at least 1 major and 1 minor criterion or at least 3 minor criteria) or if pathological data established a diagnosis of IE [21]. The recorded complications included any organ dysfunction or clinical deterioration that was considered by the authors to be related to the IE. The quality of evidence of included studies’ outcomes was assessed using the Grading of Recommendations Assessment, Development and Evaluation (GRADE) [22].

### 2.5. Statistical Analysis

Data are presented as number (%) for categorical variables and median (interquartile range, IQR) or mean (±standard deviation, SD) for continuous variables. The above-mentioned statistics were calculated with GraphPad Prism 6.0 (GraphPad Software, Inc., San Diego, CA, USA).

## 3. Results

### 3.1. Literature Search

**Figure 1:**
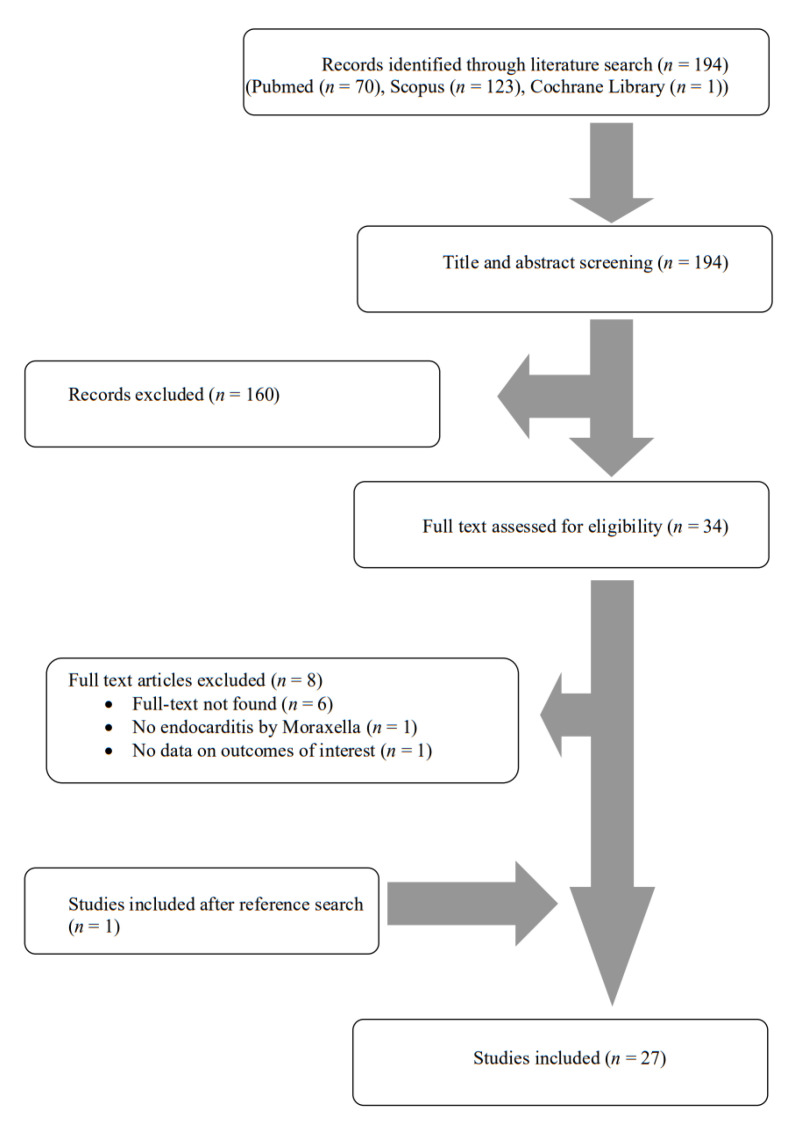
Flow diagram of study inclusion.

A total of 194 articles from PubMed, Scopus and Cochrane Library were screened. After reviewing the titles and abstracts, 34 articles were selected for full-text review. From these studies, eight were excluded from the review: six articles could not be found, one article did not describe endocarditis by *Moraxella* and one study did not report any outcomes of interest. Additionally, one study was included after reference search of the aforementioned studies. Finally, 27 met the present study’s inclusion criteria [16,17,23,24,25,26,27,28,29,30,31,32,33,34,35,36,37,38,39,40,41,42,43,44,45,46,47]. The review process is graphically presented in Figure 1.

### 3.2. Included Studies’ Characteristics

The 27 studies that were finally included in the present analysis involved 31 patients in total. Supplementary Table S1 summarizes the characteristics of the included studies. Among those studies, 11 were conducted in North and South America, 8 in Asia, 7 in Europe, and 1 in Oceania. There were 25 case reports and 2 case series; thus, the overall quality of the evidence that contributed to this systematic review was rated as very low [22].

### 3.3. Epidemiology of IE by Moraxella spp.

Age of patients ranged from six months to 77 years, the mean age was 43.4 years, and 51.6% (16 out of 31 patients) were male. Patients younger than 18 years were 12.9% (4 out of 31). A prosthetic cardiac valve was present in 25.8% (8 out of 31 patients) and was bioprosthetic in 60% (3 out of 5 patients with available data) and metallic in 40% (2 out of 5 patients). There were no patients with a recent history of otitis, pneumonia or COPD exacerbation. Table 1 shows the characteristics of patients with IE by *Moraxella* spp.

### 3.4. Microbiology and Antimicrobial Resistance of IE by Moraxella spp.

Isolated species included *M. lacunata* in 38.7% (12 out of 31 patients), *M*. *catarrhalis* in 22.6% (7 patients), *M. nonliquefaciens* in 16.1% (5 patients), *M. osloensis* in 9.7% (3 patients), *M. phenylpyruvica* in 9.7% (3 patients) and *M. liquefaciens* in 3.2% (1 patient). Isolated species were identified in blood cultures in 93.5% (29 out of 31 patients) and in valve culture in 6.5% (2 patients). Identification was performed with API in 37.5% (6 out of 16 patients with available data), 16s-rRNA in 31.3% (5 patients), ID32 GN in 25% (4 patients) and MALDI-TOF in 18.8% (3 patients). *Moraxella* spp. were resistant to penicillin in 26.7% (4 out of 15 strains) and resistant to ampicillin in 12.5% (2 out of 16 strains). None was resistant to cephalosporins (0 out of 20 strains).

### 3.5. Diagnosis of IE by Moraxella spp.

The most common site of infection was the mitral valve in 58.3% (14 out of 24 patients) and the aortic valve in 50% (12 patients). In 8.3% (2 patients), multiple valves were infected. Diagnosis was facilitated by transthoracic echocardiography in 35.5% (11 out of 31 patients), and transesophageal echocardiography in 25.8% (8 patients), while diagnosis was set at autopsy in 3.2% (1 out of 31 patients) and with valve culture and histology in 3.2% (1 patient). In 32.3% (10 out of 31 patients), diagnosis was made on empirical manner due to lack of echocardiographic data. However, in all cases, diagnosis was confirmed with the current modified Dukes’ diagnostic criteria by this study’s investigators.

### 3.6. Clinical Characteristics of IE by Moraxella spp.

Duration of symptoms varied widely; however, 82.6% (17 out of 23 patients with available data) had a duration of symptoms that was up to a month before presentation. Median duration of symptoms was 14 days, and IQR was 4 to 30 days. Fever was present in 87.1% (27 out of 31 patients), sepsis in 65.4% (17 out of 26 patients with available data), embolic phenomena in 22.6% (7 out of 31 patients), heart failure in 19.4% (6 out of 31 patients), immunologic phenomena occurred in 19.4% (6 out of 31 patients) and septic shock in 10% (3 out of 30 patients). Furthermore, 19.4% (6 out of 31 patients) developed a paravalvular abscess.

### 3.7. Treatment and Outcomes of IE by Moraxella spp.

Treatment administered for IE by Moraxella spp. was intravenous in all cases and can be seen in detail in Supplementary Table S1 and in summary in Table 1. Duration of treatment among survivors ranged from 3 to 8 weeks, with a median duration of 6 weeks. Combination treatment with an aminoglycoside was performed in 43.3% of patients (13 out of 30 with available data). A comparison of patients treated with combination therapy with those that were treated with monotherapy is shown in Table 2. Surgical management along with antimicrobials was performed in 29% (9 out of 31 patients). Overall mortality was 12.9% (4 out of 31 patients) and the mortality attributed directly to IE was 9.7% (3 patients). One patient died due to thrombus in the left ventricle postoperatively. Among patients that had surgery, overall mortality was 22.2% (2 out of 9 patients) and among patients that did not have surgery, overall mortality was 9.1% (2 out of 22 patients).

## 4. Discussion

In this study, we described the characteristics of patients with IE caused by *Moraxella* spp. Mitral valve was the most commonly infected site, while the most common clinical presentations were fever, sepsis, embolic phenomena and heart failure. Aminoglycosides, cephalosporins, penicillin and aminopenicillin were the most frequently used antimicrobials, while 12.9% of patients died.

Among the *Moraxella* spp., the most common species that has been studied in humans, and is most commonly associated with disease, is *M. catarrhalis*. It was initially considered to be non-pathogenic, but several lines of evidence showed its pathogenic potential for the human respiratory tract [1,4,5,6,7]. For example, *M. catarrhalis* has been shown to colonize the respiratory tract of individuals, sometimes allowing the development of diseases such as otitis media, lower respiratory tract in COPD or older patients, nosocomial pneumonia, sinusitis and bacteremia [1]. Experience from infections by other species of the *Moraxella* genus is rare, with few infections being mentioned in the literature, such as keratitis, endophthalmitis, bacteremia, osteomyelitis, septic arthritis and endocarditis, even though reporting of such rare species may be highly influenced by publication bias [41,48,49,50,51,52,53].

During the last decades, there have been increasing reports of these pathogens as causes of IE. IE is a rare disease carrying significant mortality and morbidity. It is most commonly caused by Gram-positive bacteria; however, it may be caused by Gram-negative bacteria in some cases, often in the context of previous hospitalization or exposure to the healthcare system [54,55,56]. Information regarding the particularities of clinical presentation and specific guidelines on the management of IE by Gram-negative species in the literature is generally inadequate [12,13,56]. For this reason, better understanding of IE caused by different species, especially Gram-negative bacteria, is required in a systematic way to identify any differences in terms of their epidemiology, clinical presentation, treatment and outcomes. More specifically, IE by *Moraxella* spp. is a very rare disease with evidence in the literature being scarce. To our knowledge, this is the first study to systematically review IE by *Moraxella* spp. and provide data on its epidemiology, microbiology, clinical characteristics, treatment and outcomes.

The mean age of patients diagnosed with *Moraxella* spp. IE in this study was 43.4 years, which is within the reported age range of diagnosis of IE by other non-HACEK Gram-negative microorganisms in the literature (40 to 70 years) [55,57,58,59,60,61,62,63,64]. However, the age at diagnosis of IE in general cohorts of IE patients is higher, and is close to 70 years [14,65,66,67]. There was a slight male predominance among patients with IE by *Moraxella* spp., while a male predominance was also noted in IE by other non-HACEK Gram-negative microorganisms and in other cohorts of patients with IE in the general population [14,15,55,57,58,59,60,61,62,63,64,65,67]. A prosthetic valve was present in 25.8% of patients with IE by *Moraxella* spp., which was a rate similar to the rate noted in other studies of IE by non-HACEK Gram-negative bacteria (14% to 59%) and similar to the rate noted in the cohorts of patients diagnosed with IE in general [55,57,58,59,60,61,62,63,64,65,66,67]. Rheumatic fever was noted in the past medical history of patients diagnosed with IE by *Moraxella* spp. in 9.7%, which is similar to the rate in other studies of patients with IE in general [66,67].

The most commonly infected intracardiac sites were the mitral valve in 58.3% and the aortic valve in 50%. This is in accordance with other studies with IE by non-HACEK Gram-negative bacteria, where the mitral valve was the most commonly infected valve in 31% to 58% of people, followed by the aortic valve in 17% to 33% [55,61,63]. In other studies, however, the aortic valve was the most commonly infected valve in 33.3% to 45%, followed by the mitral valve in 26.7% to 40% [59,60,62], or the tricuspid valve in 33% [57]. In studies with IE in the general population, however, aortic valve was the most commonly infected valve, followed by the mitral valve in most studies [65,67].

Regarding clinical presentation, the most common symptom was fever, which occurred in 87.1% of patients, while 65.4% of patients developed sepsis and 10% developed septic shock. In other studies with IE by non-HACEK Gram-negative bacteria, fever was present in 90.5% to 100% of patients [55,57,59,60,61,62,63], and sepsis was noted in 39% to 84.6% [59,60,61,62,63], while septic shock was noted in 3.4% [64]. In patients with IE in general, fever was present in 84% and shock was diagnosed in 9% [65,66]. Diagnosis of heart failure was performed in 19.4% of patients with IE by *Moraxella* spp., which is similar to that in cases of non-HACEK Gram-negative IE that ranged from 8% to 37% [55,57,59,60,61,62,63,64] and lower to the rate noted in IE in general, which was between 33% and 52% [65,67]. Immunologic and embolic phenomena in IE by *Moraxella* spp. were present in 19.4% and 22.6%, respectively, which are similar to those noted in other studies with IE by non-HACEK Gram-negative bacteria, where the rates ranged from 8% to 27% and 17% to 65%, respectively [55,57,58,59,60,61,62,63,64]. In patients with IE in general, the corresponding rates were 15.9% and 15–45% [65,66]. A paravalvular abscess was found in 19.4% of patients with IE by *Moraxella* spp., which is within the rate found in other cases of IE by non-HACEK Gram-negative bacteria, which was in the range of 8% to 42% [55,57,59,60,61,62,63].

Regarding antimicrobial resistance, *Moraxella* spp., had notable resistance to penicillin and somewhat lower resistance to aminopenicillins; however, susceptibility to cephalosporins was 100%.

In this review, overall mortality was 12.9%, with the vast majority of them due to the IE. This rate is comparable to the one noted in other studies of IE by non-HACEK Gram-negative bacteria, where mortality was as high as 43.8%, and at the lower range of mortality noted in other studies with patients with IE in general, where mortality was within the range 11–40% [14,15,55,57,58,59,60,61,62,63,64,65,66,67].

This systematic review has some limitations that should be noted. Firstly, it mainly consists of case reports. For this reason, these results should be read cautiously, since the quality of evidence that is presented by these studies is low. Moreover, the possibility of publication bias may have affected the presented data. However, since there is no original study with an adequate number of patients giving information on IE by *Moraxella* spp., we could not have used another methodology to study IE by *Moraxella* spp.

## 5. Conclusions

To conclude, this systematic review describes the epidemiology, microbiology, clinical characteristics, treatment and outcomes of IE by *Moraxella* spp. Cephalosporins, aminoglycosides, aminopenicillins and penicillin were the antimicrobials that were most commonly used.

## Figures and Tables

**Table 1:** Characteristics of 31 patients with Infective Endocarditis by *Moraxella* spp. Values show cases among patients with available data.

Characteristic	Value (*n* out of 31, Unless Otherwise Mentioned)
Male, *n* (%)	16 (51.6%)
Age, mean (SD) in years	43.4 (21.3)
Predisposing factors	
Prosthetic valve, *n* (%)	8 (25.8%)
Bad teeth hygiene or recent dental work, *n* (%)	6 (19.4%)
Congenital heart disease, *n* (%)	4 (12.9%)
Rheumatic fever, *n* (%)	3 (9.7%)
Previous IE, *n* (%)	2 (6.5%)
ESRD on hemodialysis, *n* (%)	2 (6.5%)
Recent cardiac surgery (within three months), *n* (%)	2 (6.5%)
Valve localization	
Mitral valve, *n* (%)	14 out of 24 (58.3%)
Aortic valve, *n* (%)	12 out of 24 (50%)
Multiple valves, *n* (%)	2 out of 24 (8.3%)
Method of diagnosis	
Transthoracic echocardiography, *n* (%)	11 (35.4%)
Transesophageal echocardiography, *n* (%)	8 (25.8%)
Empirical diagnosis, *n* (%)	10 (32.3%)
Autopsy, *n* (%)	1 (3.2%)
Clinical characteristics	
Fever, *n* (%)	27 (87.1%)
Sepsis, *n* (%)	17 out of 26 (65.4%)
Embolic phenomena, *n* (%)	7 (22.6%)
Heart failure, *n* (%)	6 (19.4%)
Paravalvular abscess, *n* (%)	6 (19.4%)
Immunologic phenomena, *n* (%)	6 (19.4%)
Septic shock, *n* (%)	3 out of 30 (10%)
Treatment	
Duration of treatment in weeks, median (IQR)	6 (6–6)
Aminoglycoside, *n* (%)	13 out of 30 (43.3%)
Cephalosporin, *n* (%)	16 out of 30 (53.3%)
Penicillin, *n* (%)	8 out of 30 (26.7%)
Aminopenicillin, *n* (%)	8 out of 30 (26.7%)
Carbapenem, *n* (%)	1 out of 30 (3.3%)
Quinolone, *n* (%)	1 out of 30 (3.3%)
Macrolide, *n* (%)	1 out of 30 (3.3%)
Chloramphenicol, *n* (%)	1 out of 30 (3.3%)
Surgical management, *n* (%)	9 (29%)
Outcomes	
Deaths due to infection, *n* (%)	3 (9.7%)
Deaths overall, *n* (%)	4 (12.9%)

ESRD: end-stage renal disease; IE: Infective Endocarditis; IQR: interquartile range; SD: standard deviation.

**Table 2:** Characteristics of patients with Infective Endocarditis by *Moraxella* spp. in regard to antimicrobial treatment. Values show cases among patients with available data.

Characteristic	Antibiotic Regimen Including Aminoglycosides (*n* = 13)	Antibiotic Regimen without Aminoglycosides (*n* = 17)
Male, *n* (%)	8 out of 13 (61.5%)	8 out of 17 (47.1%)
Age, mean (SD) in years	44.3 (20.1)	42.6 (23.4)
Predisposing factors		
Prosthetic valve, *n* (%)	4 out of 13 (30.8%)	4 out of 17 (23.5%)
Bad teeth hygiene or recent dental work, *n* (%)	4 out of 13 (30.8%)	2 out of 17 (19.4%)
Congenital heart disease, *n* (%)	1 out of 13 (7.7%)	3 out of 17 (17.6%)
Rheumatic fever, *n* (%)	2 out of 13 (15.4%)	1 out of 17 (5.9%)
Previous IE, *n* (%)	0 out of 13 (0%)	2 out of 17 (11.8%)
ESRD on hemodialysis, *n* (%)	1 out of 13 (15.4%)	1 out of 17 (5.9%)
Recent cardiac surgery (within three months), *n* (%)	0 out of 13 (0%)	2 out of 17 (11.8%)
Valve localization		
Mitral valve, *n* (%)	6 out of 11 (54.5%)	8 out of 12 (66.7%)
Aortic valve, *n* (%)	6 out of 11 (54.5%)	5 out of 12 (41.7%)
Tricuspid valve, *n* (%)	0 out of 11 (0%)	0 out of 12 (0%)
Multiple valves, *n* (%)	1 out of 11 (9.1%)	1 out of 12 (8.3%)
Clinical characteristics		
Fever, *n* (%)	12 out of 13 (92.3%)	14 out of 17 (82.4%)
Sepsis, *n* (%)	9 out of 12 (75%)	7 out of 13 (53.8%)
Embolic phenomena, *n* (%)	2 out of 13 (15.4%)	5 out of 17 (29.4%)
Heart failure, *n* (%)	2 out of 13 (15.4%)	4 out of 17 (23.6%)
Septic shock, *n* (%)	1 out of 12 (8.3%)	1 out of 17 (5.9%)
Immunologic phenomena, *n* (%)	3 out of 13 (23.1%)	3 out of 17 (17.6%)
Paravalvular abscess, *n* (%)	3 out of 13 (23.1%)	2 out of 17 (11.8%)
Treatment		
Duration of treatment in weeks, median (IQR)	6 (6–6)	6 (6–6)
Cephalosporin, *n* (%)	8 out of 13 (61.5%)	8 out of 17 (47.1%)
Penicillin, *n* (%)	3 out of 13 (23.1%)	5 out of 17 (29.4%)
Aminopenicillin, *n* (%)	3 out of 13 (23.1%)	5 out of 17 (29.4%)
Macrolide, *n* (%)	0 out of 13 (0%)	1 out of 17 (5.9%)
Chloramphenicol, *n* (%)	0 out of 13 (5.9%)	1 out of 17 (5.9%)
Carbapenem, *n* (%)	0 out of 13 (0%)	1 out of 17 (5.9%)
Quinolone, *n* (%)	0 out of 13 (0%)	1 out of 17 (5.9%)
Surgical management, *n* (%)	3 out of 13 (23.1%)	5 out of 17 (29.4%)
Outcomes		
Deaths due to infection, *n* (%)	1 out of 13 (7.7%)	2 out of 17 (11.8%)
Deaths overall, *n* (%)	1 out of 13 (7.7%)	3 out of 17 (17.6%)

IE: Infective Endocarditis; IQR: interquartile range; SD: standard deviation.

## Data Availability

The data presented in this study are available on request from the corresponding author.

## Supplementary Materials

The following supporting information can be downloaded at: https://www.mdpi.com/article/10.3390/jcm11071854/s1, Table S1: Characteristics of the included studies.
