# Supplementary material for: Infective Endocarditis by Moraxella Species: A Systematic Review
Source: J Clin Med. 2022 Mar 27;11(7):1854. doi: 10.3390/jcm11071854 (PMC8999714; doi:10.3390/jcm11071854)
Supplement: Supplementary file 1 [file jcm-11-01854-s001.zip › jcm-1579112-supplementary.pdf]

Table S1. Characteristics of the included studies.

Study, year published	Number of patients	Age (years)	Gender	Site of infection, n (%)	Microbiology of infection, n (%)	Treatment administered	Infection Outcomes, n (%)
Silberfarb et al, 1968 [23]	1	77	Male	AoV 1 (100)	*M. liquefaciens* 1 (100)	Aminopenicillin 1 (100)	Clinical cure[a] 0 (0)<br>Deaths overall 1 (100)<br>Deaths due to IE 1 (100)
Hewstone et al, 1968 [24]	1	12	Female	NR 1 (100)	*M. lacunata* 1 (100)	Penicillin 1 (100)<br>Aminopenicillin 1 (100)<br>Macrolide 1 (100)	Clinical cure 1 (100)<br>Deaths

						Chloramphenicol 1 (100)	overall 0
Douer et al, 1977 [25]	1	45	Female	NR 1 (100)	*M. catarrhalis* 1 (100)	Penicillin 1 (100)	Clinical cure 1 (100) Deaths overall 0
Bechard et al, 1979 [26]	1	50	Male	MV 1 (100)	*M. nonliquefaciens* 1 (100)	Aminopenicillin 1 (100) Aminoglycoside 1 (100)	Clinical cure 1 (100) Deaths overall 0
Chen et al, 1982 [27]	1	32	Female	NR 1 (100)	*M. nonliquefaciens* 1 (100)	Penicillin 1 (100)	Clinical cure 1 (100) Deaths overall 0
Turner et al,	1	62	Female	AoV 1 (100)	*M. catarrhalis* 1	Penicillin 1 (100)	Clinical cure

1985 [28]					(100)	Surgical management 1 (100)	0 (0)<br>Deaths overall 0 (0)<br>Deaths due to IE 1 (100)
Sanyal et al, 1990 [29]	1	4.5	Female	AoV 1 (100)	*M. lacunata* 1 (100)	Penicillin 1 (100)<br>Aminoglycoside 1 (100)	Clinical cure 1 (100)<br>Deaths overall 0
Periyakoil et al, 1996 [30]	1	19	Female	MV 1 (100)	*M. catarrhalis* 1 (100)	Cephalosporin 1 (100)	Clinical cure 1 (100)<br>Deaths overall 0
Guttigoli et	1	40	Male	NR 1 (100)	*M. phenylpyruvica*	Cephalosporin 1 (100)	Clinical cure

al, 2000 [31]					1 (100)	Aminoglycoside 1 (100)	1 (100) Deaths overall 0
Stefanou et al, 2000 [17]	1	37	Male	MV 1 (100)	*M. catarrhalis* 1 (100)	Aminopenicillin 1 (100)	Clinical cure 1 (100) Deaths overall 0
Tripodi et al, 2002 [32]	1	50	Male	AoV 1 (100)	*M. phenylpyruvica* 1 (100)	Cephalosporin 1 (100) Aminoglycoside 1 (100)	Clinical cure 1 (100) Deaths overall 0
Nagano et al, 2003 [33]	1	1.25	Male	NR 1 (100)	*M. lacunata* 1 (100)	Carbapenem 1 (100)	Clinical cure 1 (100) Deaths

							overall 0
Millat et al, 2003 [34]	1	45	Female	AoV 1 (100)	*M. phenylpyruvica* 1 (100)	NR 1 (100)<br>Surgical management 1 (100)	Clinical cure 1 (100)<br>Deaths overall 0
Maayan et al, 2004 [16]	4	30, 30, 34, 45	4 Male	AoV 1 (25)<br>MV 1 (25)<br>NR 2 (50)	*M. lacunata* 4 (100)	Penicillin 1 (25)<br>Aminopenicillin 1 (25)<br>Cephalosporin 2 (50)<br>Aminoglycoside 3 (75)	Clinical cure 4 (100)<br>Deaths overall 0
Kutlesa et al, 2007 [35]	1	60	Female	MV 1 (100)	*M. lacunata* 1 (100)	Penicillin 1 (100)<br>Aminoglycoside 1 (100)<br>Surgical management 1 (100)	Clinical cure 1 (100)<br>Deaths overall 0
Noman et al,	1	42	Male	MV 1 (100)	*M. lacunata* 1	Penicillin 1 (100)	Clinical cure

2008 [36]					(100)		1 (100) Deaths overall 0
Akinyemi et al, 2010 [37]	1	67	Female	MV 1 (100)	*M. nonliquefaciens* 1 (100)	Cephalosporin 1 (100) Surgical management 1 (100)	Clinical cure 1 (100) Deaths overall 0
Callejo et al, 2010 [38]	1	34	Female	MV 1 (100)	*M. lacunata* 1 (100)	Cephalosporin 1 (100) Aminoglycoside 1 (100) Surgical management 1 (100)	Clinical cure 1 (100) Deaths overall 0
Rafiq et al, 2011 [39]	1	64	Female	AoV 1 (100)	*M. nonliquefaciens* 1 (100)	Cephalosporin 1 (100) Quinolone 1 (100)	Clinical cure 1 (100) Deaths

							overall 0
Dasari et al, 2011 [40]	1	32	Female	AoV 1 (100)<br>MV 1 (100)	*M. lacunata* 1 (100)	Cephalosporin 1 (100)<br>Aminoglycoside 1 (100)	Clinical cure 1 (100)<br>Deaths overall 0
Nakayama et al, 2014 [41]	1	74	Female	MV 1 (100)	*M. lacunata* 1 (100)	Cephalosporin 1 (100)<br>Aminoglycoside 1 (100)	Clinical cure 1 (100)<br>Deaths overall 0
Shahani et al, 2015 [42]	1	63	Male	AoV 1 (100)	*M. catarrhalis* 1 (100)	Cephalosporin 1 (100)	Clinical cure 1 (100)<br>Deaths overall 0
Gagnard et al,	2	51, 75	Male	AoV 1 (50)	*M. osloensis 2*	Aminopenicillin 1 (50)	Clinical cure

2015 [43]				MV 1 (50)	(100)	Cephalosporin 1 (50)<br>Aminoglycoside 1 (50)<br>Surgical management 2 (100)	2 (100)<br>Deaths overall 0
Duployez et al, 2017 [44]	1	62	Male	AoV 1 (100)	*M. nonliquefaciens* 1 (100)	Aminopenicillin 1 (100)<br>Cephalosporin 1 (100)<br>Aminoglycoside 1 (100)	Clinical cure 0 (0)<br>Deaths overall 1 (100)<br>Deaths due to IE 1 (100)
Paiva et al, 2018 [45]	1	41	Male	MV 1 (100)	*M. osloensis* 1 (100)	Aminopenicillin 1 (100)<br>Cephalosporin 1 (100)<br>Surgical management 1	Clinical cure 1 (100)<br>Deaths

						(100)	overall 0
Maierean et al, 2019 [46]	1	65	Female	MV 1 (100)	*M. catarrhalis* 1 (100)	Cephalosporin 1 (100)	Clinical cure 1 (100) Deaths overall 0
Ahmed et al, 2020 [47]	1	0.5	Female	AoV 1 (100) MV 1 (100)	*M. catarrhalis* 1 (100)	Cephalosporin 1 (100) Surgical management 1 (100)	Clinical cure 0 (0) Deaths overall 1 (100) Deaths due to IE 1 (100)

[a] Defined as clinical resolution of the infection as a result of treatment.

AoV: aortic valve; MV: mitral valve
